# Herbal slimming formulations or remedies interact with antiretroviral therapy

**DOI:** 10.4102/sajhivmed.v18i1.742

**Published:** 2017-04-25

**Authors:** Mukesh Dheda

**Affiliations:** 1 Pharmacovigilance Centre for Public Health Programmes, National Department of Health, South Africa; 2 School of Health Science, University of KwaZulu-Natal, South Africa

Concurrent use of vitamins, supplements and herbs among people on antiretroviral therapy to help manage the side effects or improve their general health is very common in South Africa. Among these are various slimming herbs.

Many slimming products are complex mixtures of herbs that contain organic compounds that may induce or inhibit drug metabolising enzymes (e.g. cytochrome P450) and drug transporters (e.g. P-glycoprotein). These interactions may reduce or increase serum antiretroviral drug concentrations. Subtherapeutic antiretroviral drug levels in patients lead to suboptimal viral control whilst increased concentration may lead to higher risk of toxicity.^1,2^ These products may also contain compounds that interfere with the absorption of the antiretrovirals.

There are limited data from scientifically sound studies assessing the interaction between herbal slimming products and antiretroviral therapy. Recently, during the pharmacovigilance training conducted by the National Department of Health Pharmacovigilance Centre for Public Health across South Africa, healthcare professionals have expressed concerns regarding the safety of herbal slimming preparations when used concomitantly with antiretrovirals.

Healthcare professionals are requested to be vigilant and report any change in the patient’s condition when using slimming preparations concomitantly with antiretrovirals, especially when they are co-administered. Please complete adverse drug reaction forms where adverse reactions are suspected.

Adverse drug reaction forms can be obtained from the National Department of Health Pharmacovigilance Centre for Public Health Programmes. Any comments and queries can be addressed to the below email:

Tel: +27 (0)12 395 9506/8099

Fax to email: 086 241 2473

Email: npc@health.gov.za
